# Dietary patterns of university students in the UK: a cross-sectional study

**DOI:** 10.1186/s12937-018-0398-y

**Published:** 2018-10-05

**Authors:** E. F. Sprake, J. M. Russell, J. E. Cecil, R. J. Cooper, P. Grabowski, L. K. Pourshahidi, M. E. Barker

**Affiliations:** 1 Human Nutrition Unit, Department of Oncology & Metabolism, The Medical School, University of Sheffield, Sheffield, S10 2RX UK; 2 Corporate Information & Computing Service, University of Sheffield, Sheffield, S10 2GU UK; 3 Medical & Biological Sciences Building, University of St Andrews, St Andrews, KY16 9TF UK; 4 School of Health and Related Research, University of Sheffield, Sheffield, S1 4DA UK; 5 Nutrition Innovation Centre for Food & Health (NICHE), Ulster University, Coleraine, BT52 1SA UK; 6 Food & Nutrition Group, Business School, Sheffield Hallam University, Sheffield, S1 1WB UK

**Keywords:** Food consumption, Principal components analysis, Survey, University students

## Abstract

**Background:**

University represents a key transition into adulthood for many adolescents but there are associated concerns about health and behaviours. One important aspect relates to diet and there is emerging evidence that university students may consume poor quality diets, with potential implications for body weight and long-term health. This research aimed to characterise dietary patterns of university students in the UK and their sociodemographic and lifestyle antecedents.

**Methods:**

An online, cross-sectional survey was undertaken with a convenience sample of 1448 university students from five UK universities (King’s College London, Universities of St Andrews, Southampton and Sheffield, and Ulster University). The survey comprised a validated food frequency questionnaire alongside lifestyle and sociodemographic questions. Dietary patterns were generated from food frequency intake data using principal components analysis. Nutrient intakes were estimated to characterise the nutrient profile of each dietary pattern. Associations with sociodemographic variables were assessed through general linear modelling.

**Results:**

Dietary analyses revealed four major dietary patterns: ‘vegetarian’; ‘snacking’; ‘health-conscious’; and ‘convenience, red meat & alcohol’. The ‘health-conscious’ pattern had the most favourable micronutrient profile. Students’ gender, age, year of study, geographical location and cooking ability were associated with differences in pattern behaviour. Female students favoured the ‘vegetarian’ pattern, whilst male students preferred the ‘convenience, red meat & alcohol’ pattern. Less healthful dietary patterns were positively associated with lifestyle risk factors such as smoking, low physical activity and take-away consumption. The health-conscious pattern had greatest nutrient density. The ‘convenience, red meat & alcohol’ pattern was associated with higher weekly food spending; this pattern was also identified most consistently across universities. Students reporting greater cooking ability tended towards the ‘vegetarian’ and ‘health-conscious’ patterns.

**Conclusions:**

Food intake varied amongst university students. A substantial proportion of students followed health-promoting diets, which had good nutrient profiles obviating a need for dietary intervention. However, some students consumed poor diets, incurred greater food costs and practised unfavourable lifestyle behaviours, which may have long-term health effects. University policy to improve students’ diets should incorporate efforts to promote student engagement in cooking and food preparation, and increased availability of low cost healthier food items.

**Electronic supplementary material:**

The online version of this article (10.1186/s12937-018-0398-y) contains supplementary material, which is available to authorized users.

## Background

University students represent a substantial proportion (50%) of the UK young adult population [1] and an individual’s university career may be influential in the establishment of long-term eating patterns and thus chronic disease risk. This population also represents a group of young adults with a set of unique factors driving dietary intake: the transition to university life may be associated with increased autonomy over food choice, small food budgets, and exposure to new social groups and food cultures.

A limited body of data indicates that the dietary behaviours of UK university students are not conducive to either short- or long-term health. Alcohol consumption has received most research attention revealing that binge drinking is endemic [2, 3]. There are also indications of high intakes of confectionery and fast foods, and low consumption of fruit and vegetables [3, 4]. Although there is some evidence that dietary behaviours track from adolescence to adulthood [5, 6], the transition from home to university life has been associated with unfavourable changes to food intake: increases in alcohol and sugar intake, and decreases in fruit and vegetable consumption have been reported [7].

Additionally, the first year of university life has been identified as a period associated with body weight gain in both North American [8] and UK students [9, 10]. Such weight gain may have long-term repercussions, since overweight during young adulthood has been identified as a significant predictor of obesity later in life [11]. Furthermore, high rates of body dissatisfaction and dieting behaviours have been noted, particularly amongst female students [12, 13]. Such engagement in dieting behaviour and dysfunctional relationships with food not only impact on dietary adequacy [14, 15], but may also create tension and conflict for young people as they develop relationships with new peer groups [16].

Dietary studies of British university students are constrained by crude dietary assessment, small sample size and generally focus on a single university [3, 4]. Furthermore, their analytical approach has been on single foods and/or nutrients, which has allowed assessment of intake relative to dietary recommendations. Using multivariate statistical techniques to identify dietary patterns through intake of multiple interrelated food groups captures the complexity and multidimensional nature of diet, which is representative of real life food consumption [17]. This approach also allows greater insight into the different patterns of food consumption that naturally occur within a population and facilitates identification of sub-groups who may be most in need of health promotion efforts. Universities in particular may represent a setting in which dietary behaviours are open to change and large groups of young adults can be reached, representing an appropriate target for health promotion efforts. A dietary patterns approach has been used widely in various UK population groups, but has not been employed to characterise the diets of university students.

This study aimed to identify dietary patterns that exist within a UK university student population, to assess the nutritional profile of these patterns, and to examine socio-demographic and lifestyle variables underpinning these patterns.

## Methods

### Study design

This cross-sectional study involved a convenience sample of five regionally and socio-economically diverse universities throughout the UK (Universities of: Sheffield, Ulster, King’s College London (KCL), Southampton and St Andrews). These universities had responded positively to an invitation to participate in the research study; contact was made via university Human Nutrition or Health Sciences departments. A web-survey, comprising a validated food frequency questionnaire (FFQ) (Tinuviel Software Ltd., Warrington, UK) was used to assess dietary intake. Socio-demographic and lifestyle data were also collected. The survey was conducted between Autumn 2013 and Spring 2015. Data collection was preceded by a pilot study, which was used to refine the web-survey.

Ethical approval was obtained from each participating university. Informed consent for participation was obtained on the first page of the web-survey.

### Subjects & recruitment

All British and European Union students less than 30 years of age at the five participating universities represented eligible participants. A cut-off of 30 years was chosen in order to focus on the dietary behaviours of young adults. International students (non-Home or non-EU) were not included because of possible heterogeneity in food choice (this issue was identified in the pilot study), and the dietary assessment instrument used was Euro-centric. Students identifying as international students on the first page of the online survey could not proceed. Only health sciences students were recruited at the University of Southampton, because of logistical issues in distribution of the survey. All students were recruited through university email distribution lists. This email provided study details and emphasised that students did not have to be eating a healthy diet to participate. Participants were required to recall their habitual diet over the most recent university semester (three months). This was the autumn semester 2013 for students at Sheffield, the autumn semester 2014 for students at Ulster and KCL, and the spring semester 2014 for students at Southampton and St Andrews. Participants who provided their contact details were entered into a prize draw; each person could win one of 40 £20 high street vouchers.

### Participant eligibility

**Fig. 1:**
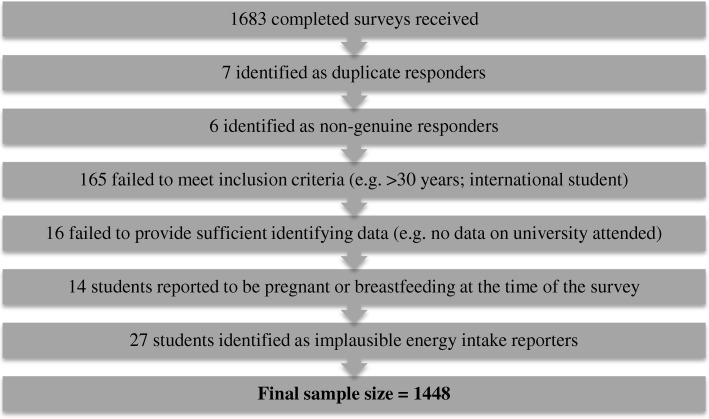
Numbers of students excluded based on fulfilment of various eligibility criteria

A total of 1683 students across the five universities responded to the survey. Figure 1 shows numbers of students excluded based on fulfilment of various eligibility criteria. The cut-offs for implausible energy intakes in the Nurses’ Health Study (< 500 Kcal/day and > 3500 Kcal/day) and Healthcare Professionals’ Follow-up Study (< 800 Kcal/day or > 4200 Kcal/day) were used to identify and exclude participants reporting implausible energy intakes the current study. Using this method, 24 participants were identified as over-reporters (8 males; 16 females) and three participants were identified as under-reporters (1 male; 2 females). A total of 1448 students comprised the final sample.

### Dietary data

A validated 111-item FFQ originally developed by the Medical Research Council was employed to assess dietary intake (DietQ; Tinuviel Software Ltd., Warrington, UK; [18, 19]. The FFQ was piloted among 40 students at the University of Sheffield. Feedback from the pilot study led to three further items being incorporated into the questionnaire (consumption of hummus; tofu; water).

Frequencies of consumption in the questionnaire were expressed as follows: every day = 7/week, through to once per week = 1/week; once every 2–3 weeks (F) = 0.5/week; rarely/never (R) = 0. Where absolute quantities of consumption were given, these were converted into number of portions consumed per day. Food and nutrient intakes were generated directly from these FFQ data using the nutritional analysis software QBuilder (Tinuviel Software, Warrington, UK). The original 111 foods/food groups listed in the FFQ were condensed into 55 broader foods/food groups for dietary patterns analysis. These 55 foods/food groups are detailed in Additional file 1: Table S1.

### Socio-demographic, anthropometric and lifestyle data

The following socio-demographic information was collected: age; gender; degree programme and year of study; full/part-time study; nature of term-time residence; ethnicity; religion; socioeconomic status (SES); maternal education; and university attended. Information on dieting/weight loss behaviour, supplement use, cooking ability (four response options from ‘able to cook wide range of meals from raw ingredients’ through to ‘unable to cook at all’), smoking status (students were asked to self-identify as a never smoker, ex-smoker, social smoker or regular smoker), self-reported physical activity levels (students were required to self-identify as not very active, moderately active or very active), body weight (kg) and height (m) (for calculation of body mass index (BMI), kg/m^2^), cooking behaviours (consumption of: meals made from raw ingredients; pre-prepared foods; ready meals and take-aways; and meals from university cafeteria) and weekly food expenditure (£) was also collected.

### Identification of dietary patterns

To generate dietary patterns, the 55 food/food group intake variables were entered into a principal component analysis (PCA) and a varimax (orthogonal) rotation was performed. The number of components retained was determined by the scree plot, parallel analysis and component interpretability [20]. Food/food groups with factor loadings > 0.32 were used to interpret each dietary pattern.

### Statistical analysis

Pearson’s product moment correlation coefficients were calculated between pattern scores and absolute nutrient intakes. Partial correlation coefficients were also calculated, which adjusted for energy intake. Correlation coefficients ≥0.5 and ≤ − 0.5 were considered strong. Examination of scatter plots revealed no evidence of non-linear relationships between component scores and nutrient intakes.

General linear models (GLMs) were firstly fitted for demographic variables alone (model 1) and then with additional eating factors (model 2). Maternal education was not included in the models, since data were not available for all students. Religion was also not included due to confounding with ethnic background.

Variables were categorised into two groups for entry into a GLM: 1) demographic variables: gender, age, leisure-time physical activity, BMI, smoking, ethnicity, year of study, term-time accommodation, university attended, and full-time/part-time status 2) cooking- and eating-related variables: cooking ability, animal food consumption, frequency of consumption of meals prepared using raw ingredients, frequency of consumption of meals using pre-prepared foods, frequency of consumption of ready-meals and take-aways, frequency of consumption of meals from university cafeteria, frequency of skipping breakfast, frequency of skipping lunch, and amount spent on food.

For each retained dietary component a GLM was fitted with demographic variables only (Group 1). A second GLM was then fitted, which included significant demographic variables and variables from Group 2. Multi-comparison post-hoc tests with Sidak correction were carried out to aid interpretation of significant factors in the GLM. The Statistical Package for the Social Sciences (SPSS) Version 20 was used for all statistical analyses. A *p* value of < 0.05 was considered significant.

## Results

### Participant characteristics

The sociodemographic characteristics of the sample are shown in Table 1. The sample comprised 1064 (73.5%) women and 384 (26.5%) men. The majority of students were White British (*n* = 911; 62.9%) and registered for full-time study (*n* = 1394; 96.3%). The mean age of the sample was 21.5 years (SD 2.63 years). The majority of respondents were from the University of Sheffield (*n* = 567; 39.2%), Ulster University in Northern Ireland (*n* = 443; 30.6%) and KCL (*n* = 305; 21.1%). The remaining students were from the Universities of Southampton (*n* = 79; 5.5%) and St Andrews, Scotland (*n* = 54; 3.7%). Just over one-third of students were studying a health-related degree. The majority of students (*n* = 1000; 69.1%) reported a healthy BMI (18.5–24.99 kg/m^2^); mean BMI was 22.8 kg/m^2^ (SD 4.64 kg/m^2^).

**Table 1:** Socio-demographic characteristics of the sample

	Number	Percentage (%)^a^
Gender		
Male	384	26.5
Female	1064	73.5
Age (years)		
17–21	873	60.3
22–25	412	28.5
26–30	163	11.3
BMI (kg.m^−2^)		
< 18.5	112	7.7
18.5–24.9	1000	69.1
25–29.9	220	15.2
≥30	76	5.2
Leisure-time physical activity		
Not very active	473	32.7
Moderately active	748	51.7
Very active	227	15.7
University attended		
University of Sheffield	567	39.2
Ulster University	443	30.6
KCL	305	21.1
University of Southampton	79	5.5
University of St Andrews	54	3.7
Faculty of study		
Arts	252	17.4
Social science	285	19.7
Engineering	109	7.5
Science	212	14.6
Medicine and health	521	36.0
Full or part time status		
Full time	1394	96.3
Part time	54	3.7
Year of study		
1st year undergraduate	489	33.8
2nd year undergraduate	301	20.8
3rd year undergraduate	264	18.2
4th or higher year undergraduate	136	9.4
Postgraduate	245	16.9
Other	13	0.9
Term-time residence		
University catered accommodation	58	4.0
University self-catered accommodation	340	23.5
Private accommodation with other friends/students	610	42.1
Private accommodation on own	63	4.4
With parents/relatives	205	14.2
With partner	107	7.4
With parents/partner & children	48	3.3
With children only	9	0.6
Other	8	0.6
Ethnic background		
White British	911	62.9
White Irish	235	16.2
Other White ethnicity	139	9.6
Mixed ethnicity	45	3.1
Asian/Asian British	69	4.8
Black/African/Caribbean/Black British	15	1.0
Other	16	1.1
Would rather not say	18	1.2
Mother’s level of education		
CSE	80	5.5
Vocational	59	4.1
O Level	184	12.7
A Level	96	6.6
Degree	342	23.6
Would rather not say	120	8.3
Not asked^b^	567	39.2
Smoking habits		
Never smoker	1090	75.3
Ex-smoker	72	5.0
Social smoker	192	13.3
Regular smoker	94	6.5

^a^where percentages do not total 100% this is due to missing data

^b^This question was not available for University of Sheffield students

In terms of eating behaviours of the sample, just under two-thirds of students described themselves as regular meat-eaters, whilst approximately 10% of students identified themselves as vegetarian. Just over half (55%) of students reported that they were able to cook a wide range of meals from raw ingredients, and 73% consumed self-cooked meals from raw ingredients ‘every’ or ‘most’ days. One in four students reported that they consumed meals cooked from pre-prepared foods, which could be assumed to represent convenience foods, ‘most days’ or ‘everyday’. Approximately 30% of students reported that they skipped breakfast at least most days. Just less than one quarter of students spent less than £20 on food each week; a weekly food budget of £20–29 was most common. Almost one in five students spent over £40 on food each week. Full details are provided in tabular form in Additional file 1: Table S2).

### Dietary patterns

Four principal components were retained, which explained 21.7% of the total variance in food intake. The first component explained 8.4% variance; the three remaining components explained 5.7%, 4.2% and 3.4% of the variance in food intake respectively. Table 2 shows the factor loadings of each of the food groups in the four dietary components retained.

**Table 2:** Factor loadings of the 55 food groups in the four principal components extracted from the PCA of frequency of food intake data of 1448 university students

Food group (% variance)	Vegetarian (8.4%)	Snacking (5.7%)	Health-conscious (4.2%)	Convenience, red meat & alcohol (3.4%)
Pulses, beans & lentils	**0.642**	− 0.113	0.216	
Tofu	**0.627**			0.105
Meat alternatives	**0.586**	0.126	− 0.109	0.121
Hummus	**0.585**		0.147	
Chicken/poultry	*−0.456*		0.106	0.277
Processed meat	*−0.453*	0.277		**0.354**
Red meat & offal	*−0.439*	0.163	0.134	**0.332**
Biscuits, cakes & sweets		**0.623**		− 0.106
Milk & cream-based desserts		**0.531**	0.160	
Confectionery	− 0.174	**0.524**		
Crisps & savoury snacks		**0.413**	−0.170	0.253
White bread	−0.141	**0.393**	−0.209	0.214
Fruit juice		**0.354**		
Other bread	0.104	**0.342**		
Canned fruit	0.101	**0.320**	0.100	−0.124
Fruit squash (not low calorie)		0.293	−0.182	
Other yogurts		0.276	0.216	−0.105
Other spread		0.251		
Added sugar in tea, coffee & cereal		0.239		0.128
Quiche	0.201	0.218		0.124
Fatty fish & canned tuna	−0.120		**0.616**	
White fish & shell fish	−0.157		**0.531**	
Nuts	**0.324**		**0.491**	
Eggs	−0.151	− 0.120	**0.477**	**0.350**
Fresh fruit	0.174		**0.443**	−0.108
Other green vegetables, onions & salad items	**0.369**	−0.258	**0.376**	0.127
Oat- & bran-based breakfast cereals		−0.172	**0.372**	−0.170
Herbal & green tea	0.313	−0.153	**0.365**	
Low fat & low-calorie yogurts			**0.334**	−0.308
Tea & coffee		0.122	0.251	
Fried food				**0.503**
Pasta & rice	0.135			**0.451**
Ready-made sauces				**0.396**
Pizza		**0.327**	−0.171	**0.392**
Chips	− 0.160	0.301	− 0.221	**0.379**
Alcoholic drinks				**0.328**
Butter	−0.166	0.137		0.312
Mayonnaise, salad cream & other dressings	−0.115	0.249	0.225	0.277
Cream		0.128	0.198	0.209
Crispbread	0.144		0.132	−0.179
Peas			0.115	
Boiled, mashed, roast & jacket potatoes	−0.211	0.261		0.113
Root vegetables & sweetcorn	0.237		0.300	
Baked beans		0.112		0.112
Wheat bran			0.124	− 0.136
Low calorie squash & fizzy drinks		0.115		
Non-white bread				
Low fat, olive & pufa spread			−0.124	
Fizzy drinks (not low calorie)	−0.180	**0.332**	− 0.204	0.282
Jam, marmalade & honey		0.255		−0.125
Cheese	0.214	0.145		0.218
Water		−0.253	0.292	
Milk	−0.162	0.107	0.120	0.106
Other breakfast cereals	−0.150	0.168	− 0.194	
Soups	0.209	0.125	0.215	

Food groups with factor loadings ≥0.10 & ≤ − 0.10 are displayed; those ≥0.32 are highlighted in bold and those ≤ − 0.32 are italicised

The first dietary component had high positive factor loadings (≥ 0.32) for pulses, beans and lentils, tofu, meat alternatives, hummus, nuts, and other green vegetables and salad items. It had high negative factor loadings for poultry, processed meat, and red meat and offal. This dietary pattern was labelled ‘vegetarian’, because there was a clear tendency towards consumption of non-meat protein sources and avoidance of all meat and fish products. The second dietary component had high positive factor loadings for biscuits, cakes and sweet pastries, milk- and cream-based desserts, confectionery, crisps and savoury snacks, fruit juice, other bread, pizza and fizzy drinks. This component was labelled ‘snacking’, because it was mainly characterised by snack-type foods that generally did not represent components of main meals, require no preparation and offered many options for mobile consumption. The third component had high positive factor loadings for fatty fish and canned tuna, white- and shellfish, nuts, eggs, fresh fruit, other green vegetables and salad items, oat- and bran-based breakfast cereals, herbal and green tea, and low fat/low calorie yogurts. This dietary pattern was labelled ‘health-conscious’, because it was characterised by foods typically associated with improved health, and was congruent with dietary components labelled ‘health-conscious’ or ‘prudent’ in other dietary pattern studies [21]. Finally, the fourth component was labelled ‘convenience, red meat & alcohol’, because it had high factor loadings for red meat and savoury foods requiring little or no preparation, and it was the only component with a positive loading on alcoholic drinks. There were also high factor loadings for fried food, pasta and rice, ready-made sauces, pizza, chips, alcoholic drinks, processed meat, red meat and offal, and eggs; there was a strong negative factor loading for low fat/low calorie yogurts.

### Correlational analyses

Pearson’s correlation coefficients between dietary pattern scores and energy intake were calculated. These are displayed in Table 3. There was a weak negative correlation between the ‘vegetarian’ pattern and energy intake (*r* = − 0.096; *p* <  0.01), but a weak positive correlation between the ‘health-conscious’ pattern and energy intake (*r* = 0.271; *P* <  0.01). The ‘snacking’ and ‘convenience, red meat and alcohol’ dietary patterns exhibited the strongest correlations with energy intake (*r* = 0.582 and *r* = 0.547 respectively). Owing to these significant associations, energy-adjusted nutrient intakes were used to explore relationships with dietary patterns scores. There were strong positive correlations (0.5 ≥ *r* < 0.6; *p* < 0.01) between the ‘vegetarian’ pattern and energy-adjusted intakes of fibre, copper and thiamin. The ‘health-conscious’ pattern was the most nutrient dense, with significant, positive, strong correlations (0.5 ≥ *r* < 0.7; *p* <  0.01) for energy-adjusted intakes of selenium, vitamin D, vitamin B12, and biotin. The ‘snacking’ pattern was strongly positively correlated with energy-adjusted non-milk extrinsic sugars (NMES) (*r* = 0.524; *P* < 0.01). Alcohol intake (energy-adjusted) was negatively correlated with scores on the ‘snacking’ pattern (*r* = − 0.317; *P* < 0.01). Only intake of total sugars (energy-adjusted) was strongly and negatively correlated with the ‘convenience, red meat & alcohol’ pattern (*r* = − 0.577; *P* < 0.01).

**Table 3:** Pearson’s correlations between dietary pattern scores and estimated average daily nutrient intakes from frequency of food intake data

	Vegetarian		Snacking		Health-conscious		Convenience, red meat & alcohol	
Nutrient	Absolute	Adjusted	Absolute	Adjusted	Absolute	Adjusted	Absolute	Adjusted
Energy (kcal)	−0.096 ^γ^		**0.582** ^γ^		0.271 ^γ^		**0.547** ^γ^	
Protein (g)	−0.304 ^γ^	− 0.389 ^γ^	0.309 ^γ^	− 0.343 ^γ^	0.483 ^γ^	0.469 ^γ^	0.491 ^γ^	0.334 ^γ^
Total fat (g)	− 0.171 ^γ^	− 0.183 ^γ^	**0.602** ^γ^	0.232 ^γ^	0.291 ^γ^	0.116 ^γ^	**0.535** ^γ^	0.134 ^γ^
Total carbohydrate (g)	0.073 ^γ^	0.322 ^γ^	**0.633** ^γ^	0.316 ^γ^	0.101 ^γ^	− 0.287 ^γ^	0.330 ^γ^	− 0.358 ^γ^
NMES (g)	−0.163 ^γ^	− 0.110 ^γ^	**0.696** ^γ^	**0.524** ^γ^	− 0.124 ^γ^	− 0.393 ^γ^	0.234 ^γ^	− 0.174 ^γ^
Saturated fat (g)	− 0.266 ^γ^	− 0.326 ^γ^	**0.638** ^γ^	0.347 ^γ^	0.166 ^γ^	− 0.098 ^γ^	0.485 ^γ^	0.080 ^γ^
Monounsaturated fat (g)	− 0.241 ^γ^	− 0.306 ^γ^	**0.558** ^γ^	0.144 ^γ^	0.302 ^γ^	0.142 ^γ^	**0.507** ^γ^	0.091 ^γ^
Polyunsaturated fat (g)	0.018 ^γ^	0.143 ^γ^	0.430 ^γ^	−0.026	0.336 ^γ^	0.209 ^γ^	0.492 ^γ^	0.137
Total sugars (g)	0.019	0.123 ^γ^	**0.602** ^γ^	0.333 ^γ^	0.295 ^γ^	0.154 ^γ^	0.043	**−0.577** ^γ^
Fibre (g)	0.443 ^γ^	**0.551** ^γ^	0.080 ^γ^	−0.259 ^γ^	0.386 ^γ^	0.306 ^γ^	0.096 ^γ^	−0.207 ^γ^
Sodium (mg)	0.113 ^γ^	0.286 ^γ^	0.439 ^γ^	−0.002 ^γ^	0.313 ^γ^	0.172 ^γ^	0.436 ^γ^	0.040 ^γ^
Potassium (mg)	0.035	0.196 ^γ^	0.360 ^γ^	−0.240 ^γ^	0.472 ^γ^	0.451 ^γ^	0.352 ^γ^	−0.212 ^γ^
Calcium (mg)	0.073 ^γ^	0.183 ^γ^	0.449 ^γ^	0.106 ^γ^	0.315 ^γ^	0.189 ^γ^	0.199 ^γ^	−0.258 ^γ^
Magnesium (mg)	0.229 ^γ^	0.461 ^γ^	0.253 ^γ^	−0.347 ^γ^	**0.509** ^γ^	0.482 ^γ^	0.304 ^γ^	− 0.197 ^γ^
Iron (mg)	0.147 ^γ^	0.332 ^γ^	0.247 ^γ^	−0.350	0.339 ^γ^	0.214	0.400 ^γ^	− 0.017
Copper (mg)	0.343 ^γ^	**0.545** ^γ^	0.229 ^γ^	−0.256 ^γ^	0.458 ^γ^	0.387 ^γ^	0.340 ^γ^	− 0.035
Zinc (mg)	−0.264 ^γ^	−0.318 ^γ^	0.289 ^γ^	− 0.382 ^γ^	0.391 ^γ^	0.304 ^γ^	0.483 ^γ^	0.080 ^γ^
Selenium (mg)	−0.221 ^γ^	− 0.208 ^γ^	0.208 ^γ^	− 0.259 ^γ^	**0.584** ^γ^	**0.555** ^γ^	0.423 ^γ^	0.115 ^γ^
Iodine (μg)	− 0.260 ^γ^	− 0.247 ^γ^	0.259 ^γ^	− 0.065	**0.524** ^γ^	0.488 ^γ^	0.126 ^γ^	−0.224 ^γ^
Vitamin A (μg)	0.132 ^γ^	0.163 ^γ^	0.050	− 0.129 ^γ^	0.362 ^γ^	0.314 ^γ^	0.065	− 0.095 ^γ^
Vitamin E (mg)	0.163 ^γ^	0.286 ^γ^	0.347 ^γ^	−0.022	**0.505** ^γ^	0.447 ^γ^	0.244 ^γ^	− 0.145 ^γ^
Vitamin D (μg)	−0.136 ^γ^	− 0.113 ^γ^	0.015	− 0.209 ^γ^	**0.645** ^γ^	**0.613** ^γ^	0.159 ^γ^	−0.009
Thiamin (mg)	0.484 ^γ^	**0.558** ^γ^	0.217 ^γ^	0.010	0.044	−0.059	0.200 ^γ^	0.004
Riboflavin (mg)	−0.223 ^γ^	− 0.216 ^γ^	0.338 ^γ^	− 0.090 ^γ^	0.394 ^γ^	0.298 ^γ^	0.210 ^γ^	−0.258 ^γ^
Niacin (mg)	−0.359 ^γ^	−0.429 ^γ^	0.221 ^γ^	− 0.377 ^γ^	0.465 ^γ^	0.408 ^γ^	0.408 ^γ^	0.008
Vitamin B_6_ (mg)	−0.210 ^γ^	− 0.226 ^γ^	0.266 ^γ^	− 0.435 ^γ^	0.332 ^γ^	0.199 ^γ^	0.439 ^γ^	− 0.011
Vitamin B_12_ (mg)	− 0.315 ^γ^	− 0.311 ^γ^	0.180 ^γ^	− 0.163 ^γ^	**0.583** ^γ^	**0.537** ^γ^	0.230 ^γ^	− 0.065
Folate (μg)	0.177 ^γ^	0.313 ^γ^	0.191 ^γ^	− 0.294 ^γ^	0.416 ^γ^	0.329 ^γ^	0.253 ^γ^	− 0.155 ^γ^
Biotin (μg)	0.088 ^γ^	0.169 ^γ^	0.100 ^γ^	−0.319 ^γ^	**0.690** ^γ^	**0.673** ^γ^	0.212 ^γ^	− 0.123 ^γ^
Vitamin C (mg)	0.202 ^γ^	0.244 ^γ^	0.163 ^γ^	−0.017 ^γ^	0.299 ^γ^	0.237 ^γ^	0.009	− 0.197 ^γ^
Alcohol (g)	0.023	0.064	−0.020	− 0.317 ^γ^	0.026	− 0.086 ^γ^	0.345 ^γ^	0.180 ^γ^

^γ^*P* <  0.01

Correlation coefficients between absolute nutrient intakes and relative nutrient intakes adjusted for energy intakes are both shown. Correlation coefficients ≥0.5 are highlighted in bold

### General linear models

Adjusted mean pattern scores by demographic and cooking/eating behaviour variables from the GLMs are provided in Table 4 (Model 1) and Table 5 (Model 2). The text that follows summarises the key findings.

**Table 4:** General Linear Model 1 – Demographic Variables

	Vegetarian		Snacking		Health-conscious		Convenience, red meat & alcohol	
Lack of fit	*p* = 0.612		*p* = 0.330		*p* = 0.280		*p* = 0.012	
Demographic variable	Adjusted mean pattern score	*p* value	Adjusted mean pattern score	*p* value	Adjusted mean pattern score	*p* value	Adjusted mean pattern score	*p* value
Gender								
Male	**0.082**	**< 0.001**	−0.315	0.074	0.378	0.132	**0.475**	**< 0.001**
Female	**0.304**		−0.428		0.469		**−0.117**	
Age								
17–21	**0.133** ^**a**^	**0.020**	−0.326	0.424	**0.262** ^**b**^	**0.015**	0.228	0.496
22–25	**0.339** ^**a**^		−0.429		**0.434** ^**a**^		0.210	
26–29	**0.197**		−0.361		**0.574** ^**b**^		0.100	
Leisure-time physical activity								
Not very active	**0.184** ^**a**^	**0.045**	**−0.171** ^**ab**^	**< 0.001**	**0.029** ^**ab**^	**< 0.001**	**0.250** ^**a**^	**0.032**
Moderately active	**0.308** ^**a**^		**−0.356** ^**ac**^		**0.383** ^**ac**^		**0.097** ^**a**^	
Very active	**0.177**		**−0.588** ^**bc**^		**0.857** ^**bc**^		**0.191**	
BMI								
< 18.5	0.292	0.221	−0.281	0.391	0.437	0.055	0.139	0.092
18.5–24.9	0.289		−0.436		0.407		0.073	
25–29.9	0.154		−0.432		0.574		0.144	
≥ 30	0.156		−0.339		0.275		0.361	
Smoking status								
Never	**0.086** ^**a**^	**0.025**	−0.333	0.270	0.404	0.173	**−0.026** ^**ab**^	**< 0.001**
Ex	**0.421** ^**a**^		−0.393		0.387		**0.121** ^**c**^	
Social	**0.159**		−0.254		0.562		**0.311** ^**ac**^	
Regular	**0.225**		−0.507		0.340		**0.310** ^**b**^	
Ethnicity								
White British	0.214	0.441	−0.299	0.810	**0.263** ^**a**^	**0.004**	0.206	0.585
White Irish	0.364		−0.381		**0.276** ^**b**^		0.254	
White Other	0.182		−0.322		**0.545** ^**ab**^		0.140	
Mixed	0.105		−0.352		**0.627**		0.297	
Asian/Asian British	0.281		−0.272		**0.309**		0.211	
Black/Black British	0.003		−0.274		**0.048**		−0.041	
Other	0.103		−0.705		**0.882**		0.489	
Rather not say	0.531		−0.370		**0.437**		−0.123	
Year of study								
1st year UG	0.212	0.194	−0.240	0.154	**0.477** ^**a**^	**0.041**	0.179	0.134
2nd year UG	0.080		−0.439		**0.503**		0.203	
3rd year UG	0.090		−0.475		**0.614** ^**a**^		0.139	
≥ 4th year UG	0.091		−0.431		**0.480**		0.410	
Postgraduate	0.177		−0.374		**0.282**		0.309	
Other	0.687		−0.272		**0.182**		−0.166	
Term-time accommodation								
Uni catered	0.129	0.963	**−0.104** ^**a**^	**< 0.001**	0.176	0.068	0.374	0.053
Uni self-catered	0.245		**−0.517** ^**b**^		0.236		0.219	
Private with friends	0.242		**−0.397** ^**a**^		0.341		0.201	
Private on own	0.324		**−0.265**		0.450		−0.275	
Parents/relatives	0.173		**−0.076** ^**bc**^		0.524		0.175	
Partner	0.269		**− 0.306** ^**c**^		0.456		0.187	
Parents/partner + children	0.138		**−0.247**		0.290		0.074	
Children only	0.218		**−0.555**		0.344		0.254	
Other	0.268		**−0.879**		0.992		0.402	
University								
Sheffield	**0.146** ^**abc**^	**< 0.001**	**−0.370** ^**a**^	**0.003**	**0.098** ^**abcd**^	**< 0.001**	0.166	0.270
Ulster	**−0.376** ^**adef**^		**−0.214** ^**ab**^		**0.318** ^**aef**^		0.299	
KCL	**0.398** ^**bd**^		**−0.569** ^**b**^		**0.541** ^**be**^		0.237	
Southampton	**0.227** ^**e**^		**−0.264**		**0.584** ^**cf**^		0.221	
St Andrews	**0.719** ^**cf**^		**−0.442**		**0.576** ^**d**^		−0.027	
Faculty								
Arts	0.334	0.234	−0.308	0.527	0.456	0.766	0.275	0.277
Social science	0.180		−0.357		0.464		0.191	
Engineering	0.123		−0.416		0.400		0.153	
Science	0.216		−0.453		0.357		0.177	
Medicine & health	0.261		−0.324		0.440		0.099	
Full-time vs. part-time student status								
Full-time	0.183	0.582	**−0.109**	**0.001**	0.381	0.560	0.246	0.378
Part-time	0.263		**−0.634**		0.466		0.113	

Independent associations between dietary pattern scores and non-nutrient variables. *p* values < 0.05 are highlighted in bold. Common superscript letters indicate significant post-hoc differences between categories within each variable

**Table 5:** General Linear Model 2 – Demographic + Eating related variables

	Vegetarian		Snacking		Health-conscious		Convenience, red meat & alcohol	
Lack of fit	*p* = 0.001		*p* = 0.748		*p* = 0.426		*p* = 0.017	
Demographic variable (n)	Adjusted mean pattern score	*p* value	Adjusted mean pattern score	*p* value	Adjusted mean pattern score	*p* value	Adjusted mean pattern score	*p* value
Gender								
Male	**1.119**	**< 0.001**	*Not entered into model*		*Not entered into model*	*N/A*	**0.645**	**< 0.001**
Female	**1.304**						**0.129**	
Age								
17–21	**1.140** ^**a**^	**0.020**	*Not entered into model*	*N/A*	**−0.047**	**0.049**	*Not entered into model*	*N/A*
22–25	**1.301** ^**a**^				**0.113** ^**a**^			
26–29	**1.314**				**0.161** ^**b**^			
Leisure-time physical activity								
Not very active	1.258	0.183	**0.270** ^**ab**^	**0.012**	**−0.187** ^**ab**^	**< 0.001**	0.436	0.117
Moderately active	1.297		**0.208** ^**ac**^		**0.064** ^**ac**^		0.327	
Very active	1.199		**0.034** ^**bc**^		**0.350** ^**bc**^		0.399	
BMI								
< 18.5	*Not entered into model*	*N/A*	*Not entered into model*	*N/A*	0.110	0.215	*Not entered into model*	*N/A*
18.5–24.9					0.057			
25–29.9					0.173			
≥ 30					−0.037			
Smoking status								
Never	1.190	0.292	*Not entered into model*	*N/A*	*Not entered into model*	*N/A*	**0.224** ^**ab**^	**< 0.001**
Ex	1.321						**0.272** ^**c**^	
Social	1.264						**0.520** ^**ac**^	
Regular	1.230						**0.532** ^**b**^	
Ethnicity								
White British	*Not entered into model*	*N/A*	*Not entered into model*	*N/A*	**−0.107** ^**ab**^	**0.016**	*Not entered into model*	*N/A*
White Irish					**−0.080** ^**c**^			
White Other					**0.123** ^**ac**^			
Mixed					**0.243**			
Asian/Asian British					**0.033**			
Black/Black British					**− 0.081**			
Other					**0.370** ^**b**^			
Rather not say					**0.106**			
Year of study								
1st year UG	*Not entered into model*	*N/A*	*Not entered into model*	*N/A*	**0.048** ^**a**^	**0.004**	*Not entered into model*	*N/A*
2nd year UG					**0.069**			
3rd year UG					**0.200** ^**a**^			
≥ 4th year UG					**−0.008**			
Postgraduate					**− 0.158**			
Other					**0.304**			
Term-time accommodation								
Uni catered	*Not entered into model*	*N/A*	**0.427** ^**ab**^	**0.033**	*Not entered into model*	*N/A*	**0.595**	**0.026**
Uni self-catered			**0.159** ^**ac**^				**0.495**	
Private with friends			**0.149** ^**bd**^				**0.469**	
Private on own			**0.218**				**0.030** ^**a**^	
Parents/relatives			**0.390** ^**cde**^				**0.431** ^**a**^	
Partner			**0.248** ^**e**^				**0.378**	
Parents/partner + children			**0.378**				**0.293**	
Children only			**− 0.178**				**0.430**	
Other			**−0.256**				**0.364**	
University								
Sheffield	**1.218** ^**abc**^	**< 0.001**	**0.136** ^**a**^	**0.029**	**−0.270** ^**abcd**^	**< 0.001**	*Not entered into model*	*N/A*
Ulster	**0.894** ^**adef**^		**0.242** ^**abc**^		**0.069** ^**aef**^			
KCL	**1.424** ^**bd**^		**0.036** ^**b**^		**0.196** ^**be**^			
Southampton	**1.298** ^**eg**^		**0.337**		**0.187** ^**cf**^			
St Andrews	**1.424** ^**cfg**^		**0.103** ^**c**^		**0.197** ^**d**^			
Full-time vs. part-time student status								
Full-time	*Not entered into model*	*N/A*	**0.442**	**< 0.001**	*Not entered into model*	*N/A*	*Not entered into model*	*N/A*
Part-time			**−0.101**					
Cooking/eating-related variables								
Cooking ability								
Wide range	**1.350** ^**ab**^	**0.036**	0.024	0.190	**0.257** ^**ab**^	**0.002**	0.261	0.297
Limited range	**1.239** ^**ac**^		0.015		**0.065** ^**ac**^		0.301	
Pre-prepared only	**1.125** ^**bc**^		0.151		**−0.101** ^**bc**^		0.527	
Unable to cook at all	**1.292**		0.492		**0.082**		0.459	
Animal food consumption								
Regular meat-eater	**−0.171** ^**abcd**^	**< 0.001**	0.187	0.080	**0.445** ^**a**^	**< 0.001**	**0.500** ^**ab**^	**< 0.001**
Flexitarian	**0.291** ^**aefg**^		0.199		**0.488** ^**b**^		**0.185** ^**ac**^	
Lacto-ovo	**1.635** ^**beh**^		0.314		**0.101**		**0.534** ^**c**^	
Ovo	**1.707** ^**chi**^		0.319		**−0.459** ^**ab**^		**0.201** ^**b**^	
Vegan	**2.795** ^**dghi**^		−0.238		**−0.196**		**0.517**	
Meals made from scratch								
Every day	1.322	0.136	**−0.060** ^**abc**^	**0.001**	**0.339** ^**abc**^	**< 0.001**	**0.622**	**< 0.001**
Most days	1.272		**0.146** ^**ade**^		**0.198** ^**ade**^		**0.495**	
Occasionally	1.172		**0.246** ^**bd**^		**−0.034** ^**bd**^		**0.345**	
Rarely/never	1.240		**0.350** ^**ce**^		**−0.200** ^**ce**^		**0.088**	
Meals made from pre-prepared foods								
Every day	**1.302** ^**a**^	**0.047**	**0.338** ^**a**^	**< 0.001**	**0.178** ^**ab**^	**0.002**	**0.591** ^**abc**^	**0.040**
Most days	**1.151** ^**bc**^		**0.304** ^**bc**^		**0.046** ^**acd**^		**0.336** ^**a**^	
Occasionally	**1.231** ^**bd**^		**0.143** ^**bd**^		**−0.069** ^**bce**^		**0.265** ^**b**^	
Rarely/never	**1.321** ^**acd**^		**−0.102** ^**acd**^		**0.148** ^**de**^		**0.356** ^**c**^	
Ready-meals/take-aways								
Every day	1.511	0.257	**0.584** ^**ab**^	**< 0.001**	**0.273**	**0.042**	**0.552** ^**a**^	**< 0.001**
Most days	1.222		**0.290** ^**cd**^		**0.025** ^**a**^		**0.570** ^**bc**^	
Occasionally	1.130		**−0.036** ^**bd**^		**−0.068** ^**b**^		**0.302** ^**cd**^	
Rarely/never	1.143		**−0.155** ^**acd**^		**0.073** ^**ab**^		**0.125** ^**abd**^	
Meals in university cafeteria								
Every day	1.156	0.062	0.153	0.547	0.141	0.922	0.375	0.336
Most days	1.253		0.245		0.047		0.485	
Occasionally	1.311		0.170		0.069		0.372	
Rarely/never	1.286		0.115		0.046		0.317	
Skipped breakfast								
Every day	1.358	0.062	0.221	0.101	**−0.179** ^**ab**^	**< 0.001**	**0.514** ^**ab**^	**< 0.001**
Most days	1.276		0.257		**0.066** ^**c**^		**0.609** ^**cd**^	
Occasionally	1.193		0.114		**0.126** ^**ad**^		**0.307** ^**ace**^	
Rarely/never	1.179		0.091		**0.290** ^**bcd**^		**0.119** ^**bde**^	
Skipped lunch/dinner								
Every day	1.245	0.991	0.089	0.131	0.284	0.404	**0.001**	**0.012**
Most days	1.252		0.236		0.066		**0.443**	
Occasionally	1.261		0.116		−0.031		**0.503**	
Rarely/never	1.248		0.241		−0.016		**0.602**	
Amount spent on food								
< £20	1.278	0.268	0.101	0.534	**−0.171** ^**abcd**^	**< 0.001**	**0.162** ^**abcd**^	**< 0.001**
£20–29	1.269		0.146		**−0.005** ^**aef**^		**0.344** ^**aef**^	
£30–39	1.251		0.150		**0.138** ^**beg**^		**0.385** ^**b**^	
£40–49	1.333		0.264		**0.096** ^**eh**^		**0.481** ^**ce**^	
≥ £50	1.127		0.192		**0.320** ^**dfgh**^		**0.564** ^**df**^	

Independent associations between dietary pattern scores and non-nutrient variables. *p* values < 0.05 are highlighted in bold. Common superscript letters indicate significant post-hoc differences between categories within each variable

#### Pattern 1 – Vegetarian

In Model 1 (demographic variables only) female gender (*p* < 0.001), middle age group (*p* = 0.020), moderate leisure-time activity levels (*p* = 0.045) and ex-smoker status (*p* = 0.025) were independently associated with higher scores on the vegetarian dietary pattern. Attendance at Ulster University was independently associated with lower ‘vegetarian’ pattern scores (*p* < 0.001).

In Model 2 (demographic variables & food/eating related variables), female gender (p <  0.001), middle age group (p = 0.020), greatest self-reported cooking ability (*p* = 0.036), least frequent consumption of pre-prepared foods (*p* = 0.047) and lower consumption of animal products (*p* = 0.036) were independently associated with higher ‘vegetarian’ pattern scores. Attendance at Ulster University (*p* < 0.001) was independently associated with lower scores.

#### Pattern 2 – Snacking

In Model 1, low leisure-time physical activity (*p* < 0.001), attendance at Ulster University (*p* = 0.003), full time student status (*p* = 0.001) and living with parents/other relatives (*p* < 0.001) were independently associated with higher ‘snacking’ pattern scores.

In Model 2, lower leisure-time physical activity participation (*p* = 0.012), attendance at Ulster University (*p* = 0.029), living with parents/other relatives or in university catered accommodation (*p* = 0.033), and full-time student status (*p* < 0.001) were independently associated with greater pattern score. Infrequent consumption of meals prepared from raw ingredients (*p* < 0.001), and frequent consumption of pre-prepared foods (*p* < 0.001) and ready meals/take-aways (*p* < 0.001) were also independently associated with high ‘snacking’ pattern scores.

#### Pattern 3 – Health-conscious

In Model 1, ‘very active’ physical activity levels (*p* < 0.001), ‘White Other’ ethnicity (*p* = 0.004) and third year of undergraduate study (*p* = 0.041) were independently associated with higher scores on the ‘health-conscious’ pattern. Youngest age group (*p* = 0.015) and attendance at University of Sheffield were independently associated with lower scores (*p* < 0.001).

In Model 2, the five significant demographic factors identified in Model 1 remained independently associated with ‘health-conscious’ pattern scores. Additionally, reporting being ‘able to cook a wide range of meals from raw ingredients’ (*p* = 0.002), daily consumption of meals made from raw ingredients (*p* < 0.001) and pre-prepared foods (*p* = 0.002), greatest amount of money spent on food (≥50/week) (*p* < 0.001), at least occasional consumption of animal products (*p* < 0.001) and infrequent skipping of breakfast (*p* < 0.001) were independently associated with higher health-conscious pattern scores. Rare – compared to occasional or almost daily - consumption of take-aways/ready meals was associated with lower scores (*p* = 0.042).

#### Pattern 4 – Convenience, red meat & alcohol

In Model 1, male gender (*p* < 0.001), lowest leisure-time physical activity levels (*p* = 0.032), and regular/social smoking status (*p* < 0.001) were independently associated with higher scores on the ‘convenience, red meat & alcohol’ diet pattern. An independent inverse association between living alone in private accommodation and score on this pattern approached significance (*p* = 0.053).

In Model 2, higher pattern scores were independently associated with male gender (*p* < 0.001), regular/social smoking status (*p* < 0.001), most frequent consumption pre-prepared foods (*p* = 0.040), frequent consumption of ready-meals/take-aways (*p* < 0.001), frequent breakfast skipping (*p* < 0.001), regular consumption of animal products (*p* < 0.001) and greater amounts of money spent on food (*p* < 0.001). Lower scores were independently associated with living alone (*p* = 0.026) and spending less money on food (*p* < 0.001).

## Discussion

This study aimed to identify dietary patterns within a UK university student population and to delineate the socio-demographic, lifestyle and other behavioural characteristics of students favouring these patterns. Dietary patterns analysis unveiled heterogeneity in food choice with students following four major dietary patterns: ‘vegetarian’, ‘snacking’, ‘health-conscious’ and ‘convenience, red meat & alcohol’. These patterns explained approximately one fifth of the variance in food intake. Students’ gender, age, geographical location and cooking ability were associated with differences in pattern behaviour. Clustering of lifestyle risk factors with dietary patterns was also evident, with less healthful dietary patterns associated with smoking, low physical activity and take-away consumption. Students tending to the ‘convenience, red meat & alcohol’ pattern reported spending more money on food each week.

The ‘vegetarian’, ‘snacking’ and ‘health-conscious’ patterns identified here are analogous to those previously reported in adult and adolescent UK populations [22, 23]. The ‘convenience, red meat & alcohol’ pattern shares features (positive factor loadings for red meat, chips, alcohol) with a major dietary pattern (labelled drinker/social) reported among approximately 480 20–25 year olds in Northern Ireland, derived from 7-day diet history data [24].

The ‘snacking’ and ‘convenience, red meat and alcohol’ patterns have common features with published data on the food preferences of British university students [2, 4]. Existing studies allude to non-prudent consumption patterns, reporting low consumption of fruit and vegetables alongside high intakes of confectionery, alcohol, and fried, ready-made and convenience foods [2–4].

We have shown that both the ‘snacking’ and ‘convenience, red meat and alcohol’ patterns were least nutrient-dense. Indeed it is noteworthy that these two patterns were additionally positively correlated with energy intake and did not feature fruit and vegetables; dependence on such a pattern may increase risk of positive energy balance and hence weight gain. The ‘health-conscious’ pattern, which had a favourable nutrient profile - being particularly dense in micronutrients such as biotin, vitamin B12, vitamin D and selenium - is at odds with the stereotype of student eating patterns, but concurs with published research on dietary patterns among UK adults [21, 22] and a small-scale study of university students in Birmingham, UK [4].

It is of note that a vegetarian diet was the predominant pattern identified in the current study, and indeed 10% of students described themselves as vegetarian. The latter figure is less than that reported in a survey of over 3000 university students studying in Northern Ireland, which reported that 22% of students did not eat meat [3]. Although a vegetarian pattern has been described in the wider UK diet pattern literature [21–23], it was a minor component, in keeping with the low prevalence of vegetarianism among British adults nationally (3%) [25].

Whilst high rates of binge drinking have previously been documented among student populations [3, 26], and there is a popular stereotype of students as heavy drinkers, only one pattern (‘convenience, red meat & alcohol’) was high in alcoholic beverages. Furthermore students following this pattern were also more likely to smoke, have frequent consumption of take-aways and pre-prepared foods and engage in lower levels of physical activity. This clustering of behaviours is important, since the negative health outcomes associated with multiple lifestyle risk factors are greater than the sum of individual health risk behaviours [27]. Conversely students favouring more healthful dietary patterns reported greater engagement in other health-promoting lifestyle choices, including not smoking, greater participation in physical activity. Aggregation of lifestyle behaviours has previously been reported in both university student and adult populations [26–28].

Gendered food preferences were also evident, especially in relation to meat consumption. Specifically, female students favoured a ‘vegetarian’ diet, whilst male students scored highly on the ‘convenience, red meat & alcohol’ pattern. Greater meat and fast food consumption among male students has previously been reported, and vegetarianism is more prevalent amongst female students [3, 24]. Although a recent British student study observed no gender differences between eating patterns [4], this study lacked detailed dietary assessment.

Dietary preferences also varied between participating universities. Generally, students at Ulster University favoured less healthful patterns, whilst those at the Universities of Southampton, St Andrews and KCL tended towards more healthful diets. Students attending the University of Sheffield were least likely to adopt a ‘health-conscious’ dietary pattern. This gradient is congruent with national data, which indicates that the population of Northern Ireland consumes a diet of poorer quality than the UK as a whole [29]. Dietary gradients were also evident in relation to geography in a comparative study of university students from seven universities across the UK, although absence of information on specific university location limits comparison [2].

It is also possible that dietary differences observed between universities may arise because of socioeconomic gradients across universities. Missing data on social class for students at the University of Sheffield precluded adjustment for this possibility. However information from the Higher Education Statistics Agency (HESA) indicates an SES gradient between universities: a greater proportion of students at Ulster University are from manual occupational backgrounds than from KCL, Sheffield and Southampton (no data available for St Andrews) [30]. Maternal education data for Ulster, KCL, St Andrews & Southampton corroborated these differences (data for University of Sheffield not available). The wider literature consistently reports a positive association between socioeconomic status and diet quality across UK population groups [21, 23, 28]. However, the tendency for students at the University of Sheffield to score lowest on a ‘health-conscious’ diet is not in line with this explanation.

The possibility of selection bias should be considered. There were differences in recruitment method between the University of Sheffield and Ulster University (recruitment email distributed directly to all students via a global mailing list), and the other three participating sites (e.g. study advertisement on student volunteers webpage). These recruitment differences may have biased the sample towards health-motivated students at KCL, St Andrews and Southampton.

The lack of association between university attended and consumption of the ‘convenience, red meat & alcohol’ diet also deserves attention. This homogeneity suggests that this pattern is pervasive across all universities studied, substantiating popular beliefs that the diet of UK university students is one of poor quality.

This study also revealed that older students favoured more healthful dietary patterns and there was evidence of a positive linear relationship between age and scores on the ‘health-conscious’ pattern. It is possible that as students mature they become increasingly aware of the impact of dietary choices on health and well-being, and health thus becomes an increasingly important determinant of food choice. Studies among the general UK adult population report similar age effects [21, 22]. A student survey conducted in Northern Ireland reported a positive gradient in diet quality by year of study [3]. In contrast, other student-specific research has failed to detect an association between eating habits and age (or year of study), although most of these studies have not collected detailed dietary data [2, 4, 10, 26].

Finally, 45% of the current sample reported limited (or non-existent) cooking ability, being at best only able to cook a limited range of meals from raw ingredients. Students with poor cooking ability were less likely to adopt healthier (vegetarian; health-conscious) diets than their more skilled counterparts. This association has not been documented among a university student population, but corroborates associations found in several adult studies [31, 32]. No association, however, was identified between cooking ability and scores on the less healthful dietary patterns (snacking; convenience, red meat & alcohol). Whilst it is likely that students who lack culinary skills may be forced to rely on convenience foods to ensure meal provision, other factors such as time pressures and (lack of) cooking enjoyment may be more salient in determining students’ decisions around consumption of these foods [33, 34] .

### Study strengths and limitations

The current study had a number of strengths and limitations that should be acknowledged. FFQs are not optimal for the measurement of absolute dietary intake, but the use of a dietary pattern approach permitted ranking according to food group intake and so was considered appropriate. Furthermore, use of an FFQ allowed dietary intake to be captured over a 3-month semester and facilitated recruitment of a large, geographically diverse sample, albeit a convenience one. Ideally, the sampling frame would have included a greater number of universities and involved stratification by year of study, subject group and socioeconomic indices in order to give a nationally representative profile of student eating patterns. Moreover, only health-sciences students were recruited at Southampton, which may represent a source of bias.

The small number of students recruited from St Andrews may been seen as an under-representation of students from a Scottish university, but it should be noted that the total student population at St Andrews (population of around 8000 students) is much smaller than that of Sheffield, Ulster and KCL (between 25,000 and 30,000 students). It should also be noted that all dietary studies suffer from selection bias, in which more health- or diet-aware individuals choose to participate. Consequently, the prominence of the vegetarian and health-conscious dietary patterns may have been over-estimated in this study. Indeed, the BMI distributions were also biased towards healthy, in keeping with other student surveys [4, 26].

There was lack of fit in statistical models for ‘convenience, red meat and alcohol’, and ‘vegetarian’ dietary patterns. It should be noted that these models are developmental and clearly only cover some of the potential antecedents of following such patterns. Convenience, red meat, alcohol and vegetarian dietary choices are likely to be influenced by a raft of social, cultural and political factors, which have not been included in the model. For example, it is recognised that adoption of a vegetarian diet is related to concern about the environment and animal welfare, as well as for health reasons and weight management [35, 36]. Similarly, there is enormous heterogeneity in motives for drinking alcohol including coping, enhancement of social status, religious practice, personality type and alcohol availability [37, 38].

### Implications for policy and future research directions

Importantly, policy makers must recognise not all students consume poor diets at university: a large group of students consumed nutritionally favourable and health-promoting diets and do not appear in need of dietary intervention. However, students who consumed poor diets and practised unfavourable lifestyle behaviours were also identified, which may have long-term health effects. Targeted interventions towards these students are necessary. Furthermore, contemporary policy to limit red meat and alcohol consumption has greatest relevance to male students. University policy to improve students’ diets should also incorporate efforts to promote student engagement in cooking and food preparation, and increased availability of low cost healthier food items.

This study also highlights a number of future research needs. Replication of this research among a large representative sample of UK university students would be pertinent. Secondly, in light of the association between cooking ability and dietary consumption patterns, investigation of the potential for a cooking skills intervention to improve dietary intake is warranted. Finally, the public health impact of dietary patterns and other lifestyle risk factors established during university become most important if these behaviours track forward into working adult life and represent a blueprint for long-term dietary preferences. Longitudinal research is now needed to investigate this possibility.

## Conclusion

This study provides a unique insight into the dietary patterns of UK university students along with associated nutritional content. It has identified a number of antecedents of both healthful and unhealthful dietary practices. Four patterns emerged, with evidence of more healthful dietary practices amongst female and older students, and those with greater self-reported cooking ability. Students in Northern Ireland appeared to favour less healthful dietary patterns than those in Great Britain. Male students tended towards a diet founded on convenience food, red meat and alcohol; this pattern was germane to all participating universities. These findings are relevant to future health promotion interventions and behaviour change in this important population.

## Additional file

Additional file 1: **Table S1.** Details of the constituent foods comprising the 55 foods/food groups entered into the PCA. **Table S2.** Eating behaviours and other eating-related characteristics of the Phase 1 sample. (DOCX 26 kb)
