# Supplementary material for: Dietary patterns of university students in the UK: a cross-sectional study
Source: Nutr J. 2018 Oct 5;17:90. doi: 10.1186/s12937-018-0398-y (PMC6172790; doi:10.1186/s12937-018-0398-y)
Supplement: Supplementary file 1 — Table S1. Details of the constituent foods comprising the 55 foods/food groups entered into the PCA. Table S2. Eating behaviours and other eating-related characteristics of the Phase 1 sample. (DOCX 26 kb) [file 12937_2018_398_MOESM1_ESM.docx]

**Additional file 1**

**Table S1: Details of the constituent foods comprising the 55 foods/food groups entered into the PCA**

| F**ood groups entered into the PCA** **(n = 55)** | **Original food groups from the FFQ (n = 111)** |
| --- | --- |
| **White bread** | White bread |
| **Non white bread** | Brown, 50/50 or wheatgerm bread  Wholemeal bread or chapatis |
| **Other bread** | Other bread (e.g. rolls, teacakes, crumpets, etc) |
| **Crispbread (etc.)** | Crispbread, ryvita or cream crackers |
| **Jam, marmalade & honey (i.e. on toast)** | Jam, marmalade or honey on bread |
| **Oat/bran based breakfast cereal** | Bran flakes or sultana bran  Porridge or ready brek  All bran |
| **Other breakfast cereal** | Cornflakes  Sugar-or chocolate coated cereal (e.g. frosties, coco pops etc)  Rice krispies or Special K  Muesli, fruit & fibre or Cheerios  Weetabix, wheatflakes or shredded wheat |
| **Wheat bran** | Wheat bran |
| **Red meat & offal** | Beef (roast, steak, stewed, burgers, lasagne, bolognese, chilli, curry)  Lamb (roast, chops, stews, curry)  Pork (roast, chops, stewed, sweet & sour)  Liver, kidney, heart |
| **Chicken & other poultry** | Chicken/other poultry (roast, casserole, curry, sweet & sour) |
| **Processed meat (including meat pies & sausage rolls etc.)** | Bacon  Ham or gammon (including consumption in composite dishes)  Canned meat (e.g. corned beef), pate or meat spread  Sausages  Meat pie, pastie, sausage roll, samosa - shop bought  Meat pie, pastie, sausage roll, samosa - homemade |
| **White fish & shell fish** | White fish (cod, haddock, plaice, fish fingers, fish cakes)  Shellfish (e.g. prawns) |
| **Fatty fish & canned tuna** | Kipper, herring, mackerel, trout (including canned)  Pilchards, sardines, salmon (including canned)  Tuna (including canned) |
| **Potatoes (boiled, roast, mashed, jackets)** | Boiled or mashed potatoes  Jacket potatoes  Roast potatoes |
| **Chips** | Shop bought chips, oven chips, hash browns  Home-cooked chips |
| **Peas** | Peas |
| **Other green vegetables, onions, salad or tomatoes** | Other green vegetables, salad or tomatoes  Onions (raw, cooked, pickled) |
| **Root vegetables & sweetcorn** | Carrots  Parsnips, swedes, turnips or sweetcorn |
| **Baked beans** | Baked beans |
| **Pulses, beans (non-baked) & lentils** | Butter beans, broad beans or red kidney beans  Lentils, chick peas or dahl |
| **Pasta & rice** | Spaghetti, other pasta, noodles  Rice |
| **Quiche** | Quiche |
| **Pizza** | Pizza |
| **Meat alternatives** | Vegetarian burgers/sausages  Dishes made with TVP (soya mince) or Quorn |
| **Tofu** | Tofu |
| **Hummus** | Hummus |
| **Biscuits, cakes & sweet pastries** | Digestive biscuits/plain biscuits  Other sweet biscuits  Fruit cake/sponge cake/sponge pudding - shop bought  Fruit cake/sponge cake/sponge pudding - homemade  Fruit tart, jam tart, doughnut, danish pastry - shop bought  Fruit tart, jam tart, doughnut, danish pastry - homemade |
| **Confectionery** | Chocolate (e.g. Galaxy, Mars Bar, Twix, Kit Kat)  Sweets (e.g. fruit gums, pastilles, mints) |
| **Crisps & savoury snacks** | Crisps/savoury snacks (e.g. Quavers& tortilla chips) |
| **Nuts** | Nuts |
| **Milk- and cream-based desserts** | Ice cream, iced dessert, fool, mousse, trifle  Milk pudding (e.g. rice/tapioca/macaroni) |
| **Low fat / low calorie yogurts** | Low fat yogurt  Low calorie yogurt (e.g. Shape) |
| **Other yogurts** | Other yogurts / fromage frais |
| **Canned fruit** | Fruit canned in syrup  Fruit canned in juice |
| **Fresh fruit** | Apples  Pears  Oranges or grapefruit  Bananas  Other fruit (e.g. melon, strawberries, kiwi, grapes, peach/nectarine) |
| **Eggs** | Eggs |
| **Milk** | Milk |
| **Cream** | Cream |
| **Cheese** | Cheese (excluding cottage cheese)  Cottage cheese |
| **Butter** | Butter |
| **Low fat/olive/pufa spread** | Polyunsaturated margarine/spread  Olive oil spread  Very low fat spread (25% fat)  Low fat spread - other  Low fat spread - polyunsaturated |
| **Other spread** | Other soft margarine/spread (not olive)  Hard margarine |
| **Food that is fried** | Food that is fried (e.g. fish/onions/mushrooms/tomatoes/eggs) |
| **Tea & coffee** | Tea (non-herbal/non-green)  Coffee |
| **Herbal / green tea** | Herbal or green tea |
| **Added sugar (on cereal or toast)** | Honey or sugar on cereal  Sugar/honey in coffee/tea |
| **Fruit juice** | Fruit juice |
| **Fruit squash (not low calorie)** | Fruit squash (not low calorie) |
| **Fizzy drinks (not low calorie)** | Fizzy drinks (not low calorie) |
| **Low calorie squash & fizzy drinks** | Low calorie squash/fizzy drinks |
| **Water** | Water |
| **Alcoholic drinks** | Beer/larger/stout  Cider  Wine  Sherry/port/vermouth  Spirits/liqueurs |
| **Soups** | Vegetable-based soups  Cream of' soups |
| **Sauces (ready-made)** | Sauces (e.g. curry, sweet & sour) |
| **Mayonnaise, salad cream & other dressings** | Mayonnaise  Salad cream  Other dressings (e.g. French/thousand island/blue cheese) |
| *Not included as a food group/part of a food group for entry into the PCA* | Bread eaten dry  Fat on meat |

**Table S2: Eating behaviours and other eating-related characteristics of the Phase 1 sample**

|  | | **Number** | **Percentage (%)^γ^** |
| --- | --- | --- | --- |
| **Consumption of animal foods** | Regular meat eater  Occasional consumption of meat/poultry/fish  Avoids all meat/poultry/fish but consumes eggs & dairy  Avoids all meat/poultry/fish/eggs but consumes dairy  Avoids all animal-derived products including honey (vegan) | 878  421  95  28  26 | 60.6  29.1  6.6  1.9  1.8 |
| **Cooking ability** | Wide range of meals from raw ingredients  Limited range of meals from raw ingredients  Can cook only using pre-prepared foods  Unable to cook at all | 797  579  51  21 | 55  40  3.5  1.5 |
| **Consumption of self-cooked meals from raw ingredients** | Every day  Most days  Occasionally  Rarely/never | 405  650  303  90 | 28  44.9  20.9  6.2 |
| **Consumption of self-cooked meals using pre-prepared foods** | Every day  Most days  Occasionally  Rarely/never | 64  313  735  336 | 4.4  21.6  50.8  23.2 |
| **Consumption of ready-meals & take-aways** | Every day  Most days  Occasionally  Rarely/never | 11  121  776  540 | 0.8  8.4  53.6  37.3 |
| **Consumption of meals at university cafeteria** | Every day  Most days  Occasionally  Rarely/never | 34  103  386  925 | 2.3  7.1  26.7  63.9 |
| **Frequency of skipping breakfast** | Every day  Most days  Occasionally  Rarely/never | 129  291  380  648 | 8.9  20.1  26.2  44.8 |
| **Frequency of skipping lunch/dinner** | Every day  Most days  Occasionally  Rarely/never | 21  104  505  818 | 1.5  7.2  34.9  56.5 |
| **Money spent on food each week** | < £20  £20-29  £30-39  £40-49  ≥£50 | 342  524  335  146  101 | 23.6  36.2  23.1  10.1  7.0 |
| ***Satisfaction with eating and dieting behaviour*** | |  |  |
| **How student feels about his/her body** | Far too thin  A little too thin  Just right  A little overweight  Very overweight | 17  117  614  623  77 | 1.2  8.1  42.4  43.0  5.3 |
| **Currently dieting to lose weight** | Yes  No | 308  1140 | 21.3  78.7 |
| **Currently dieting to bulk up/gain muscle mass** | Yes  No | 279  1169 | 19.3  80.7 |
| **Contentment with food intake** | 20%  40%  60%  80%  100% | 178  335  125  421  89 | 12.3  23.1  8.6  29.1  6.1 |
| ***Use of dietary supplements*** | |  |  |
| **Use of multivitamin supplements** | Yes  No | 243  1205 | 16.8  83.2 |
| **Use of mineral supplements** | Yes  No | 63  1385 | 4.4  95.6 |
| **Use of vitamin supplements** | Yes  No | 110  1338 | 7.6  92.4 |
| **Use of protein shakes** | Yes  No | 82  1366 | 5.7  94.3 |
| **Use of other fitness supplements** | Yes  No | 23  1425 | 1.6  98.4 |
| **Use of other dietary supplements** | Yes  No | 39  1409 | 2.7  97.3 |
| ***Major factors determining food choice*** | |  |  |
| **Cost/value for money** | Yes  No | 871  577 | 60.2  39.8 |
| **Taste/preferences** | Yes  No | 374  1074 | 25.8  74.2 |
| **Health/nutritional value** | Yes  No | 405  1043 | 28.0  72.0 |
| **Dieting value/calorie content** | Yes  No | 167  1281 | 11.5  88.4 |
| **Vegetarianism** | Yes  No | 22  1426 | 1.6  98.4 |
| **Ethical reasons** | Yes  No | 20  1428 | 1.5  98.5 |
| **Quality/freshness** | Yes  No | 98  1350 | 6.8  93.2 |
| **Ease of cooking/convenience** | Yes  No | 243  1205 | 16.8  83.2 |
| **Shelf-life of food** | Yes  No | 21  1427 | 1.5  98.5 |
| **Hunger/cravings** | Yes  No | 32  1416 | 2.2  97.8 |
| **Availability of food** | Yes  No | 45  1403 | 3.1  96.9 |
| **Time available** | Yes  No | 41  1407 | 2.8  97.2 |
| **Variety** | Yes  No | 24  1424 | 1.7  98.3 |
| **Other** | Yes  No | 152  1296 | 10.5  89.5 |

^γ^ percentages which do not total 100% is due to missing data
